# Resistin Is Increased in Periodontal Cells and Tissues: *In Vitro* and *In Vivo* Studies

**DOI:** 10.1155/2020/9817095

**Published:** 2020-01-20

**Authors:** Andressa V. B. Nogueira, Marjan Nokhbehsaim, Sema Tekin, Rafael S. de Molon, Luis C. Spolidorio, Svenja Memmert, Anna Damanaki, Andreas Jäger, Sigrun Eick, James Deschner, Joni A. Cirelli

**Affiliations:** ^1^Department of Periodontology and Operative Dentistry, University Medical Center of the Johannes Gutenberg University, Mainz 55131, Germany; ^2^Section of Experimental Dento-Maxillo-Facial Medicine, Center of Dento-Maxillo-Facial Medicine, University of Bonn, Bonn 53111, Germany; ^3^Private Practice, Istanbul, Turkey, Formerly Clinical Research Unit 208, University of Bonn, Bonn 53111, Germany; ^4^Department of Diagnosis and Surgery, School of Dentistry at Araraquara, São Paulo State University-UNESP, Araraquara, São Paulo 14801-903, Brazil; ^5^Department of Physiology and Pathology, School of Dentistry at Araraquara, São Paulo State University-UNESP, Araraquara, São Paulo 14801-903, Brazil; ^6^Department of Orthodontics, Center of Dento-Maxillo-Facial Medicine, University of Bonn, Bonn 53111, Germany; ^7^Department of Periodontology, Laboratory of Oral Microbiology, University of Bern, Bern 3012, Switzerland

## Abstract

Resistin, a proinflammatory adipokine, is elevated in many inflammatory diseases. However, little is known about its performance in periodontitis. The present study is aimed at evaluating resistin expression and synthesis in periodontal cells and tissues under inflammatory/microbial stress in addition to its effects on the periodontium. In vivo, 24 male rats were randomly divided into two groups: control and ligature-induced periodontal disease. After 6 and 12 days, animals were sacrificed to analyze gene expression of adipokines, bone loss, inflammation, and resistin synthesis. In vitro, human periodontal ligament (PDL) fibroblasts were used to evaluate the expression of resistin after inflammatory stimuli. In addition, PDL fibroblasts were exposed to resistin to evaluate its role on soft and hard tissue metabolism markers. The periodontitis group demonstrated significant bone loss, an increase in the number of inflammatory cells and vascular structures, an increase in resistin expression and synthesis, and a decrease in the expression of adiponectin, leptin, and its functional receptor. PDL fibroblasts showed a significant increase in resistin expression and synthesis in response to the inflammatory stimulus by IL-1*β*. Resistin induced an increase in cytokine expression and a decrease in the regulation of some hard tissue and matrix formation genes in PDL fibroblasts. These data indicate that resistin is produced by periodontal cells and tissues, and this effect is enhanced by inflammatory stimuli. Moreover, resistin seems to interfere with soft and hard tissue metabolism during periodontitis by reducing markers related to matrix formation and bone tissue.

## 1. Introduction

Periodontitis is a highly prevalent chronic inflammatory disease that affects around 42% of the American adult population; approximately 10% of the global population is affected by a severe type [1, 2]. It is initiated and maintained by a dysbiotic dental biofilm that leads to progressive and irreversible destruction of the tooth-supporting tissues such as gingiva, cementum, periodontal ligament, and alveolar bone, resulting, eventually, in tooth loss [3]. The inflammatory reaction triggered by the host leads to increased production and release of inflammatory mediators and proteases, which results in extracellular matrix degradation and alveolar bone loss [3, 4].

Recently, evidence has been accumulated that periodontitis is associated with some systemic conditions and diseases, such as diabetes mellitus, atherosclerosis, rheumatoid arthritis, and obesity [5–10]. The systemic low-grade inflammatory state present in obesity may be a pathomechanistic link between obesity and other chronic inflammatory diseases such as periodontitis [11]. Adipokines are cytokines with both anti- and proinflammatory functions that regulate feeding behavior, energy expenditure, and insulin resistance, and act in immunoinflammatory processes [12]. Among adipokines, resistin has been extensively evaluated to better understand its role in immunoinflammatory reactions and bone metabolism [13–18]. Serum levels of this proinflammatory adipokine are elevated in obese patients [13]. Also, high levels of resistin have been detected in the gingival crevicular fluid (GCF) of patients with obesity [14]. Interestingly, resistin levels are also increased in the GCF of individuals with periodontal disease in the absence of obesity [14, 15, 19, 20]. Thus, the current evidence shows elevated levels of resistin, suggesting that this adipokine may play a role in the etiopathology of periodontitis.

Resistin is a cysteine-rich secretory protein expressed in several human inflammatory resident cells inside and outside the adipose tissue [16]. In humans, resistin is expressed mainly by macrophages in response to bacterial and inflammatory challenge, such as lipopolysaccharides, proinflammatory cytokines, and to resistin itself, suggesting a role in inflammation [18, 21, 22]. It has been demonstrated that resistin plays a role in bone remodeling as it enhances the proliferation of preosteoblast cells, and also plays a role in osteoclastogenesis since it increases the number of differentiated osteoclasts [17]. In addition, resistin exerts an angiogenetic effect on endothelial cells [23] and has strong proinflammatory effects [18]. Since serum levels of resistin are elevated in obesity and in systemic diseases such as diabetes mellitus and rheumatoid arthritis, and are also increased in the GCF and serum of patients with periodontitis, it can be assumed that resistin increases periodontal inflammation, thereby increasing the risk of periodontal disease or impairment of periodontal healing [14, 19, 20]. However, little is known about the explicit role of resistin in the etiopathogenesis of periodontitis. Thus, considering the general importance of adipokines and their relation to inflammatory diseases, this study is aimed at obtaining more information about the involvement of resistin in periodontal infection and inflammation. For this reason, the expression and synthesis of the proinflammatory adipokine resistin were examined in periodontal cells and tissues under inflammatory stress, in addition to its effects on the periodontium in vitro and in vivo.

## 2. Materials and Methods

### 2.1. Experimental Protocol

First, we evaluated whether periodontal tissues of rats could express and produce resistin and other adipokines in the course of experimental periodontitis. The animal experimental protocol (23/2012) was approved by the local Ethical Committee on Animal Experimentation and performed in accordance with the guidelines from the National Council for the Control of Animal Experimentation (CONCEA). The study protocol also followed all recommendations of the ARRIVE guidelines [24]. An ANOVA test was used to calculate the sample number based on the results of microcomputed tomography (micro-CT) analysis performed in a pilot study. A minimum difference between the means was determined to have a statistical difference of 6.00 *μ*m with a standard deviation of 2.85 *μ*m. Considering a test power of 0.80 (1 − *β* = 0.80) and a significance level of *α* = 0.05, a minimum of six animals in each group was necessary to execute the present study. Thus, a total of 24 male adult Holtzman rats, average weight of 300 g, were maintained in the animal facilities of the School of Dentistry at Araraquara under controlled temperature (22-25°C) with a 12 h light/dark cycle. Animals were housed in plastic cages and received standard laboratory diet and water ad libitum. After 1 week of acclimatization, animals were randomly divided into two experimental groups: control (sham-operated) and ligature-induced periodontal disease. At baseline, animals from the periodontitis group received general anesthesia by intramuscular injections of 10% ketamine chlorhydrate (0.08 mL/100 g body weight) and 2% xylazine chlorhydrate (0.04 mL/100 g body weight). Cotton thread ligatures were placed around the cervical area of both upper first molars and knotted mesially to induce experimental periodontal disease. After 6 and 12 days, all animals were sacrificed by anesthetic overdose. The maxillary jaws were hemisected, and one half of the maxillae including molars with their surrounding tissues were fixed in 4% paraformaldehyde for 48 h and stored in 70% ethanol for analysis of bone resorption by micro-CT. Later, these samples (6 hemimaxillae per group and time point) were decalcified in EDTA (10%, 0.5 M) for 2 months at RT [25–27] and embedded in paraffin for histological processing for stereometric and immunohistochemical (IHC) analyses. The other half of maxillae (6 hemimaxillae per group and time point) had the gingival tissues around the maxillary first molars carefully dissected for extraction of total RNA for real-time polymerase chain reaction (RT-qPCR).

### 2.2. Cell Culture and Treatment of Human Periodontal Fibroblasts

This study and the protocols were approved by the Ethics Committee of the University of Bonn, and informed consent was obtained prior to sample collection (043/11). Human periodontal ligament (PDL) fibroblasts from up to 9 donors were isolated from periodontally healthy teeth that were extracted for orthodontic reasons. Briefly, cells were cultured in Dulbecco's minimal essential medium (DMEM, Invitrogen, Karlsruhe, Germany) supplemented with 10% fetal bovine serum (FBS, Invitrogen), 100 U/mL penicillin, and 100 *μ*g/mL streptomycin (Invitrogen) at 37°C in a humidified atmosphere of 5% CO_2_. Cells between third and fifth passage were seeded (5 × 10^4^ cells/well) on six-well culture plates and grown to 80% confluence. The medium was changed every second day. One day prior to the experiments, the FBS concentration was reduced to 1%. Cells were treated with human recombinant IL-1 beta (IL-1*β*, PromoKine, Heidelberg, Germany) for 1 day to simulate an inflammatory environment. IL-1*β* was applied at a concentration of 1 ng/mL, which is in the range of levels usually found in the GCF of periodontally diseased patients. In another experiment, PDL fibroblasts were treated with resistin to evaluate its role in soft and hard tissue metabolism. A physiological concentration of resistin (100 ng/mL) was used for PDL fibroblast stimulation in vitro [15, 28]. Untreated cells served as a control.

### 2.3. Alkaline Phosphatase Activity

In order to determine the role of resistin in potential hard tissue differentiation of PDL fibroblasts, the alkaline phosphatase (ALP) specific activity was measured as a function of the release of p-nitrophenol from a phosphatase substrate, p-nitrophenylphosphate (pNPP), at pH 10.2 and normalized to total protein in PDL fibroblast lysates in the presence or absence of resistin after 1 and 6 days. To measure intracellular ALP, cells were lysed with 0.5% Triton X-100 (Sigma, Munich, Germany) in phosphate-buffered saline (PBS, Invitrogen) on ice. The cell lysates were frozen and thawed three times to disrupt the cell membranes. Then, substrate (2 mg/mL pNPP, Sigma) was added to each sample. The absorbance was determined after 30 min of incubation at 37°C with a microplate reader (Power Wave X; BioTek Instruments, Winooski, VT, USA) at 405 nm. ALP activity was expressed as *μ*mol/10^5^ cells/min.

### 2.4. RT-qPCR

Gene expressions in samples from PDL fibroblasts as well as from rat gingival biopsies were analyzed by quantitative RT-PCR. Total RNA extraction was performed using RNeasy Mini Kit (Qiagen, Hilden, Germany) according to the manufacturer's protocol. RNA concentration was determined by spectrophotometer NanoDrop ND-2000 (Thermo Fisher Scientific, Wilmington, DE, USA), and 500 ng of total RNA was reverse-transcribed using iScript™ Select cDNA Synthesis Kit (Bio-Rad Laboratories, Munich, Germany) according to the manufacturer's instruction. Analysis of gene expression of resistin, leptin and its functional receptor, adiponectin and its receptors, IL-6, IL-8, bone morphogenetic protein 2 (BMP2), runt-related transcription factor 2 (RUNX2), osteocalcin (OCN), periostin (POSTN), collagen I (COL I), transforming growth factor-beta (TGF-*β*), and vascular endothelial growth factor (VEGF) was subsequently performed in triplicate by using QuantiTect Primers (Qiagen), SYBR Green PCR Master Mix (Bio-Rad Laboratories), and the thermocycler iCycler iQ™ Real-Time PCR Detection System (Bio-Rad Laboratories). Amplification was carried out under the following conditions: initial denaturation at 95°C for 5 min followed by 40 cycles of denaturation at 95°C for 10 s and combined annealing/extension at 60°C for 30 s. Data were analyzed using the comparative cycle threshold (CT) method with glyceraldehyde-3-phosphate dehydrogenase (GAPDH) as the housekeeping gene.

### 2.5. Enzyme-Linked Immunosorbent Assay (ELISA)

Protein levels of resistin were measured in cell-free supernatants after PDL cells were exposed or not to IL-1*β* for 1 day using a commercially available ELISA kit (RayBio Human Resistin ELISA Kit, RayBiotech, Norcross, GA, USA) according to the manufacturer's protocol. The absorbance was determined with a microplate reader (PowerWave X, BioTek Instruments, Winooski, VT, USA) at 450 nm. For normalization, cells were collected and counted using an automatic cell counter (Moelab, Hilden, Germany).

### 2.6. Micro-CT

Micro-CT analysis was performed on animal tissues to evaluate the presence of bone destruction after periodontal disease induction. The micro-CT measurements were performed in accordance with a previous study [29]. A high-resolution micro-CT imaging system Skyscan 1076 (Bruker, Kontich, Belgium) was used with the following parameters: X-ray generator at 50 kVp, beam current at 500 *μ*A, 0.5 mm aluminum filtration at an image resolution of 18 *μ*m. The images were reconstructed with the software NRecon 1.6.1.5 (Skyscan) in all three spatial dimensions, and then all the maxillae images were orientated and saved in coronal slices (2000 × 1336) utilizing software Data Viewer 1.4.3.1 (Skyscan). To measure alveolar bone volume (BV) and tissue volume (TV), a region of interest (ROI) was delineated from the root apices to the alveolar crest and from the mesial root of the first molar to the distal root of the third molar, excluding the tooth roots and PDL spaces, comprising the entire alveolar bone, using the despeckle tool (custom processing) in a software program CT Analyser 1.10.1.0 (Skyscan). The architectural parameter evaluated was bone volume under tissue volume (BV/TV, %), which represents the BV fraction.

### 2.7. Stereometric Analysis

Stereometric analysis was performed to confirm the presence of significant inflammation in periodontal tissues of animals subjected to periodontitis induction. Semiserial sections of 5 *μ*m were obtained from the tissue blocks on a mesial-distal orientation. The sections were processed for regular hematoxylin and eosin (H&E) staining, and a total of 15 sections were evaluated per tissue blocks. Each of these 15 sections was spaced 50 *μ*m from each other, in order to be representative of 750 *μ*m extension. A single and blinded examiner, who was trained and calibrated to the purpose of the experiment, performed the stereometric analysis using a point-counting technique. This point-counting technique was used to evaluate the proportion of the following structures: polymorphonuclear cells, mononuclear cells, vascular structures, and other structures on the H&E-stained sections. Also, macrophages and lymphocytes were detected and counted by stereometric analysis. For this, macrophages were considered as round and large cells that contain a central round nucleus and abundant clear cytoplasm, while lymphocytes were considered as round and small cells that contain large nucleus and small cytoplasm. One area was assessed between submarginal area and bone crest area. A 250 *μ*m^2^ square-lattice grid was constructed using the image software Adobe Photoshop CS5 (San Jose, CA, USA), and the type of structure found on the intersection of the grid lines was counted on an optical microscope (Cambridge Instruments, Buffalo, NY, EUA) under 200x magnification. The results were expressed as a percentage of the total area analyzed.

### 2.8. IHC Analysis

IHC analysis was performed to evaluate the production of resistin protein within the periodontal tissues in the furcation region of first maxillary molars. In addition, CD3- (a marker of T cells) and MAC387- (a marker of macrophages) positive cells were evaluated. Serial parasagittal sections with 5 *μ*m thickness were mounted on silanized slides Dako A/S (Glostrup, Denmark). The IHC analysis was performed according to a previous study [25]. The sections were incubated with rabbit polyclonal antibody anti-resistin (1 : 100, ab216840, Abcam, Cambridge, England), rabbit polyclonal antibody anti-CD3 (1 : 100, ab5690, Abcam), and mouse monoclonal antibody macrophage marker (1 : 50, sc66204, Santa Cruz Biotechnology, Santa Cruz, CA, USA) at 4°C overnight. Negative control sections were instead incubated with 1% PBS to assess unspecific background staining. After the incubation period, the tissue sections were washed and incubated with biotinylated immunoglobulin (Dako) at room temperature for 30 min and subsequently washed again and incubated with avidin-biotin peroxidase complex (Dako) at room temperature for 30 min. Diaminobenzidine (DAB, Dako) was used as a chromogen substrate. All sections were counterstained with Carrazi's hematoxylin. Photomicrographs of the most coronal portion of the furcation region were taken using a light microscope LEICA microsystem GmbH (Wetzlar, Germany) at 200x magnification. Cells positive for resistin, CD3, and MAC387 present in the furcation region were counted by a blinded and calibrated examiner.

### 2.9. Data Analysis

Statistical analyses were performed using a software program GraphPad Prism 5 (GraphPad Software Inc., San Diego, CA, USA). For all analyses, data were synthesized using mean values and standard deviations of the means. To determine any significant difference between groups, a *t*-test and ANOVA followed by Dunnett's test were used for the in vivo study, and a Mann–Whitney *U* test was used for the in vitro study. Significant differences were considered when *p* < 0.05.

## 3. Results

### 3.1. Pathophysiological Changes in Hard and Soft Tissues during Experimental Periodontitis

**Figure 1:**
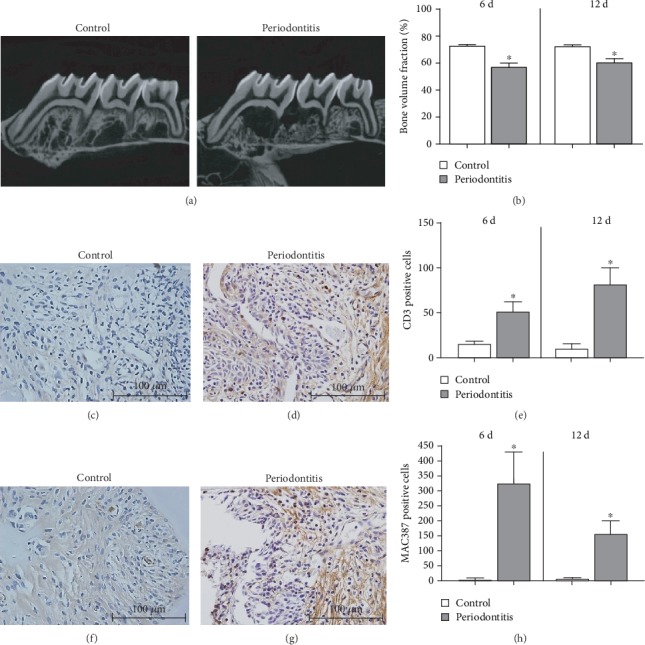
(a) Representative two-dimensional micro-CT lateral views of maxillary molars of one animal from the control group and one animal from the periodontitis group at 12 days. (b) Comparison of BVF percentage measured in a selected ROI of the maxillary first molars and the mesial roots of the second molars using micro-CT. Results are expressed as the mean ± SD (*n* = 6). The periodontitis group showed a decrease in BVF compared to the control group at 6 and 12 days. ^∗^Significant difference (*p* < 0.05). Representative images (400x magnification) showing the IHC analysis performed in the first maxillary molars of rats from the control group (c) and periodontitis group (d) marked with CD3, and the control group (f) and periodontitis group (g) marked with MAC387. Results are expressed as the mean ± SD (*n* = 6). The graphs show an increase in the number of cells positive for CD3 (e) and MAC387 (h) in the periodontitis group in comparison to the control group after 6 and 12 days. ^∗^Significant difference (*p* < 0.05).

As analyzed by micro-CT, ligature-induced periodontitis resulted in substantial alveolar bone loss (*p* < 0.05, Figures 1(a) and 1(b)). Stereometric analysis indicates that periodontal disease is associated with a sustained inflammatory reaction between submarginal area and bone crest area. Inflammation was studied by the relative presence of inflammatory cells and vascular structures in a 500 *μ*m^2^ area in a minimum of 15 sections (500 points). The density (%) of neutrophils, mononuclear cells, and vascular structures increased at day 12 for the ligature-induced periodontal disease model (Table 1). Also, the stereometrics analysis showed an increase in the number of macrophages and lymphocytes in the periodontitis group at 12 days (Table 1). IHC analysis was used to validate the inflammation found in the stereometric analysis. The result showed that in the periodontitis group, there was a significant number of cells positive for CD3 (Figures 1(c)–1(e)) and MAC387 (Figures 1(f)–1(h)) after 6 and 12 days of the disease beginning as compared to the control group.

### 3.2. Expression of Adipokines and Their Receptors in Periodontal Tissues and Cells

**Figure 2:**
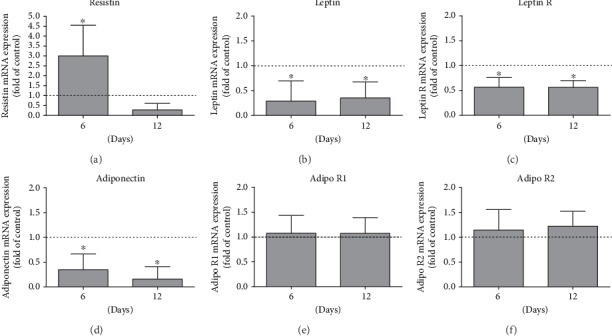
Resistin (a), leptin (b), leptin receptor (c), adiponectin (d), adiponectin receptor 1 (e), and adiponectin receptor 2 (f) mRNA expression in rat gingival biopsies after 6 and 12 days of ligature-induced experimental periodontitis around the first maxillary molars compared to expression in healthy controls. Results are expressed as the mean ± SD (*n* = 6). ^∗^Significant difference (*p* < 0.05).

**Figure 3:**
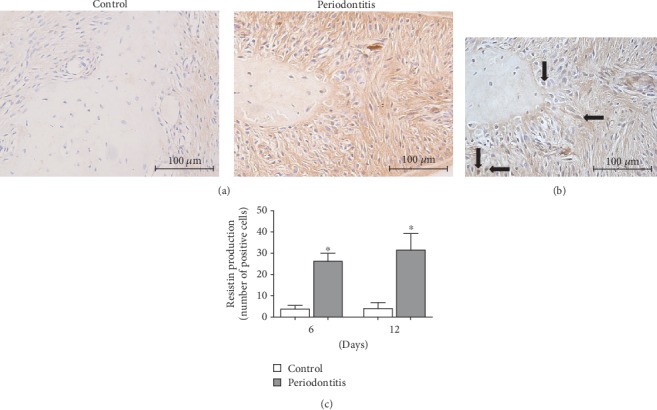
Representative images (100x magnification) of IHC staining for resistin in the first maxillary molars of rats from the control and periodontitis groups (a). Representative image (400x magnification) showing in greater scale some cells positive for resistin pointed by black arrows in the periodontitis group (b). Results are expressed as the mean ± SD (*n* = 6). The graph shows an increase in the number of cells positive for resistin in comparison to the control group (c). ^∗^Significant difference (*p* < 0.05).

When compared to the control group, the periodontitis group presented in the gingival tissue samples a significant (*p* < 0.05) increase in mRNA expression of resistin and a significant (*p* < 0.05) decrease in mRNA expression of adiponectin as well as of leptin and its functional receptor (Figures 2(a)–2(d)). Interestingly, expression of the adiponectin receptors R1 and R2 was not changed in the presence of periodontitis (Figures 2(e) and 2(f)). In addition, as detected by IHC, a significantly (*p* < 0.05) enhanced number of resistin-positive cells was found in the furcation region of the periodontitis samples as compared to the control group (Figure 3(c)).

**Figure 4:**
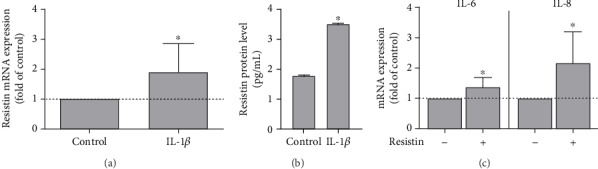
Effect of IL-1*β* (1 ng/mL) on resistin mRNA expression (a) and protein levels (b) in PDL fibroblasts after 1 day as determined by qPCR (*n* = 9/group) and ELISA (*n* = 6/group), respectively. Untreated cells served as a control. (c) Effect of resistin (100 ng/mL) on IL-6 and IL-8 mRNA expression in PDL cells (*n* = 6/group). Results are expressed as the mean ± SD. ^∗^Significant difference (*p* < 0.05).

Moreover, the in vitro experiment also showed that periodontal cells produced resistin under inflammatory conditions. Specifically, in the presence of IL-1*β*, PDL fibroblasts presented significantly (*p* < 0.05) more resistin expression (Figure 4(a)) and protein synthesis (Figure 4(b)).

### 3.3. Effect of Resistin on Critical Cell Functions of Periodontal Homeostasis

**Figure 5:**
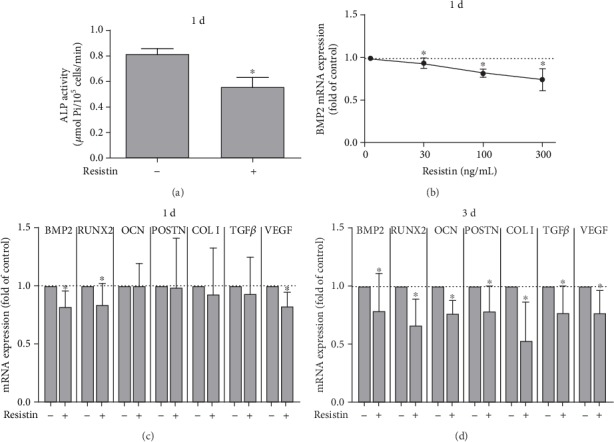
(a) Effect of resistin (100 ng/mL) on ALP activity in PDL fibroblasts after 1 day. ALP activity was significantly reduced in the presence of resistin (*n* = 3). (b) Effect of resistin (30, 100, and 300 ng/mL) on BMP2 mRNA expression in PDL fibroblasts after 1 day (*n* = 9). Effect of resistin (100 ng/mL) on hard tissue markers (BMP2, RUNX2, and OCN), matrix markers (POSTN and COL I), and growth factors (TGF-*β* and VEGF) involved in bone metabolism (*n* = 9) at 1 day (c) and 3 days (d). Results are expressed as the mean ± SD. All these molecular markers were downregulated in PDL fibroblasts after 1 and 3 days of incubation with resistin. ^∗^Significant difference (*p* < 0.05).

After having found that resistin was increased in periodontal cells and tissues under inflammatory and microbial stress, we next focused on studying the effects of resistin on human periodontal cells. These cells showed a significant increase in the expression of IL-6 and IL-8 after treatment with resistin, indicating an inflammatory effect (Figure 4(c)). Then, we sought to analyze the effect of resistin on hard tissue differentiation by evaluating the ALP activity in PDL fibroblasts incubated with resistin for 1 and 6 days. In the presence of resistin, ALP activity was significantly (*p* < 0.05) reduced at 1 d, as demonstrated in Figure 5(a). At 6 d, there was no significant difference (data not shown). A wide range of resistin concentrations caused a significant (*p* < 0.05) reduction of BMP2 mRNA expression after 1 day (Figure 5(c)). In addition, we studied the regulation of other hard tissue markers (BMP2, RUNX2, and OCN), matrix markers (POSTN and COL I), and growth factors (TGF-*β* and VEGF) involved in bone metabolism. All these molecular markers were downregulated in PDL fibroblasts after 1 and 3 days of incubation with resistin (Figures 5(c) and 5(d)).

## 4. Discussion

This study provides detailed evidence that resistin is produced locally in periodontal cells and tissues, with a remarkable increase as a result of inflammatory/infectious stress. In addition, this adipokine has negative/detrimental effects or interferes negatively with soft and hard tissue metabolism due to a decrease in matrix and bone markers and growth factors.

Interestingly, resistin has already been demonstrated to be expressed in PDL cells [30]. Our present study confirms that periodontal cells and tissues are able to produce resistin. More importantly, our study revealed that the resistin expression in PDL cells is upregulated in the presence of the proinflammatory mediator IL-1*β*. Likewise, experimental periodontitis in rats resulted in the upregulation of resistin in gingival tissues and increased resistin protein production in the periodontium. In accordance with our results, resistin has been observed in human gingival fibroblasts treated with *Porphyromonas gingivalis* and in gingival biopsies from gingivitis and periodontitis patients [31]. Moreover, our study showed a proinflammatory potential of resistin in increasing the expression of IL-6 and IL-8 in periodontal cells. Taken together, these data suggest that resistin is increased after microbial and inflammatory conditions. Furthermore, in addition to inflammatory cells such as macrophages, PDL fibroblasts may contribute to the increased resistin levels found in the GCF of periodontal patients, as we have provided not only gene expression but also protein synthesis of resistin, suggesting that resistin induces proinflammatory cytokine production in periodontal cells. Therefore, resistin may play a role in the pathology of periodontitis.

Periodontitis is a chronic inflammatory disease initiated by bacterial infection, which activates the host immune response by a cascade of inflammatory events such as the release of proinflammatory cytokines. Some studies have demonstrated elevated levels of resistin in the GCF of periodontitis patients compared to nonperiodontitis patients [15, 19, 28]. This high resistin level in the GCF during periodontitis could be explained by the quantity of PDL fibroblasts in addition to inflammatory cells, especially macrophages. As we could see, the IHC analysis of the in vivo samples showed a significant increase in the number of macrophages and lymphocytes in the periodontitis group as compared to control, which, although not further evaluated, could have been contributed to the increased resistin levels in the inflamed periodontal tissues. Moreover, as already demonstrated by other investigators, resistin stimulates the production of proinflammatory cytokines in adipose tissue and in peripheral blood mononuclear cells and, on the other hand, the expression of resistin is also stimulated by proinflammatory cytokines in the cited tissue and cells showing a positive feedback loop for resistin [18, 32, 33]. In our study, although this feedback loop was not evaluated, we could assume it is also happening in periodontal cells and tissues.

Interestingly, our study showed that resistin does interfere negatively with ALP activity by significantly reducing its expression. In addition, resistin induces downregulation of hard tissue (BMP2, RUNX2, and OCN) and matrix (POSTN and COL I) markers and growth factors (TGF-*β* and VEGF) in periodontal cells. Although limited, our results suggest an impairment effect of resistin on periodontal remodeling. For this reason, resistin could be involved in the tissue destruction presented in periodontal disease via increased expression in periodontal cells and tissues after inflammatory and infectious stimuli and by affecting the metabolism of soft and hard tissues due to inhibition of molecules involved in hard and soft tissue formation.

Evidence indicates that resistin is present in other cell types and has a role in bone metabolism. Murine preosteoclasts RAW 264.7, murine preosteoblasts MC3T3-E1, human bone marrow mesenchymal stem cells, and human osteoblasts express resistin [17]. Resistin stimulates osteoclast differentiation in both murine preosteoclasts and human monocytes and increases osteoblast proliferation. In addition, an indirect stimulatory effect of resistin on osteoclastogenesis can be observed by decreasing the RANKL/OPG mRNA ratio and by increasing IL-6 production [17]. Besides that, resistin was found in osteoblasts and osteoclasts from human osteoarthritis osteophytes. However, when these cells were stimulated with resistin, there was no significant effect on cytokine secretion [34]. Increased levels of resistin were found in human neutrophils stimulated with *P. gingivalis* LPS [15]. Also, resistin induces the production of cytokines and chemokines in human articular chondrocytes [35]. Furthermore, resistin induces the release of proinflammatory cytokines, such as IL-6 and TNF-*α*, in human peripheral blood mononuclear cells [18]. Resistin may affect directly and indirectly bone metabolism; thus, it would be interesting to evaluate the effect of resistin on osteoblasts using bone remodeling assays in the future.

Regarding the other adipokines evaluated in the present study, leptin and its functional receptor were significantly downregulated in gingival tissues in periodontitis. Our previous study conducted regarding the role of leptin in periodontal regeneration corroborates the present findings as it showed that leptin is produced by PDL cells, and bacterial/inflammatory stimuli significantly decrease leptin synthesis [36]. Furthermore, the gingival tissues showed significantly reduced expression of adiponectin in periodontitis, although no change was observed for its receptors compared to the control group. Interestingly, a study from our group showed that *P. gingivalis* suppresses adiponectin expression in PDL cells while stimulating the expression of its receptors [37]. Also, downregulation of adiponectin and increased expression of its receptors have been observed in gingival tissues of periodontal patients as compared to periodontal healthy ones [31]. All these findings are in agreement with previous studies that also found decreased levels of adiponectin in the serum of periodontitis patients [25, 38]. In contrast, gingival fibroblasts treated with TNF-*α* showed significant reduction in the expression of adiponectin receptors as well as periodontal tissues from severe periodontitis patients compared to healthy ones [39]. Thus, the exact regulation and role of adiponectin receptors in periodontitis are still contradictory.

During periodontal disease progression, many molecules induce a cascade of events to initiate and maintain a chronic immunoinflammatory response in the periodontium responsible for the destruction of soft and hard tissues [4, 40]. In the present study, the experimental periodontitis model has accomplished its objective as we could see significant alveolar bone loss in the periodontitis group in line with a significant increase in the percentage of inflammatory cells and vascular structures, and with a decrease in the extracellular matrix and fibroblast cells.

Rodent and human resistin share around 60% of the sequence homology at the amino acid level. Furthermore, their resistin genes have notably different promoter regions, suggesting that the regulation and function of resistin is different between rodents and humans [41, 42]. Therefore, whether the effect of resistin on rodents is translatable to human has not been completely confirmed. However, elevated resistin levels have been observed in serum and gingival crevicular fluid of periodontitis patients in clinical studies [14, 19, 43]. That the regulation of resistin in human and rodent periodontia might be similar is also supported by our findings which showed increased resistin expression in rat experimental periodontitis and elevated resistin levels in human PDL fibroblasts after exposure to a proinflammatory mediator. We cannot extrapolate to humans the results from animal studies; however, we can speculate after careful data analysis from a variety of animal studies and make a cross-species translation. Therefore, the quality of animal studies is extremely important to improve study design and conduction beyond to minimize biases in publication.

The cell type used in our study, PDL fibroblasts, presents a phenotype that enables it to be used for hard tissue formation analyses due to their established ability in developing mineralized nodules in vitro and to express bone-associated markers.

The concentration of resistin used in our study was in the range of the physiological concentration of resistin found in human GCF [15, 19, 28] and used to stimulate other types of cells in in vitro studies [44–46]. Also, as in our previous studies, IL-1*β* was used in vitro in order to mimic inflammatory conditions because its increased production in periodontal sites has been demonstrated [36, 40].

## 5. Conclusions

In summary, the present study shows that resistin is produced by periodontal cells and tissues. In addition, microbial and inflammatory stimuli increase resistin expression and production in the periodontium. Moreover, resistin seems to interfere with soft and hard tissue metabolism during periodontitis by reducing ALP activity and markers related to bone tissue and matrix formation. However, further studies using specific resistin inhibitors and/or knockout animals could allow a better elucidation of the harmful role of resistin in inflammatory diseases such as periodontitis.

## Figures and Tables

**Table 1:** Mean (±SD) of the percentage of polymorphonuclear cells, mononuclear cells, vascular structures, other structures, lymphocytes, and macrophages on H&E-stained sections of each experimental group.

	Control	Periodontal disease
Polymorphonuclear cells	2.57 ± 0.70	25.19 ± 5.10^∗^
Mononuclear cells	2.89 ± 1.18	51.89 ± 3.51^∗^
Vascular structures	3.12 ± 1.24	13.66 ± 2.45^∗^
Other structures	92.15 ± 9.32	9.26 ± 1.25^∗^
Lymphocytes	0.98 ± 0.46	22.71 ± 2.18^∗^
Macrophages	1.91 ± 0.67	29.18 ± 3.21^∗^

^∗^ *p* < 0.05 statistically different compared to control.

## Data Availability

The data used to support the findings of this study are available from the corresponding author upon request.
